# Quantifying the influence of water deficit on root and shoot growth in wheat using X-ray Computed Tomography

**DOI:** 10.1093/aobpla/plaa036

**Published:** 2020-07-26

**Authors:** A M Khalil, E H Murchie, S J Mooney

**Affiliations:** 1 School of Biosciences, University of Nottingham, Sutton Bonington Campus, Leicestershire, UK; 2 College of Agriculture, University of Duhok, Duhok–Kurdistan Region, Iraq

**Keywords:** Drought, photosynthesis, root architecture, stomatal conductance, water deficit, X-ray Computed Tomography

## Abstract

The potential increased frequency and severity of drought associated with environmental change represents a significant obstacle to efforts aimed at enhancing food security due to its impact on crop development, and ultimately, yield. Our understanding of the impact of drought on crop growth in terms of plant aerial tissues is much more advanced than knowledge of the below-ground impacts. We undertook an experiment using X-ray Computed Tomography that aimed to support measurements of infrared gas exchange from plant shoots with quantification of 3D root architecture traits and the associated soil structural characteristics. Winter wheat (cv. Zebedee) was assessed at two early growth stages (14 and 21 days) under four water treatments (100, 75, 50 and 25 % of a notional field capacity (FC) and across two soil types (sandy loam and clay loam)). Plants generally grew better (to a larger size) in sandy loam soil as opposed to clay loam soil, most likely due to the soil structure and the associated pore network. All plants grew poorly under extreme water stress and displayed optimal growth at 75 % of FC, as opposed to 100 %, as the latter was most likely too wet. The optimal matric potential for root and shoot growth, inferred from the water release curve for each soil type, was higher than that for photosynthesis, stomatal conductance and transpiration suggesting root and shoot growth was more affected by soil water content than photosynthesis-related characteristics under water deficit conditions. With incidences of drought likely to increase, identification of wheat cultivars that are more tolerant of these conditions is important. Studies that consider the impact of water stress on both plant shoots and roots, and the role of the soil pore system such as this offer considerable potential in supporting these efforts.

## Teaser

The likelihood of increased drought associated with environmental change represents a significant obstacle for enhancing food security due to its impact on crop development and yield. We used 3D X-ray imaging to measure root architecture traits in wheat under different drying regimes and across two soil types along with traditional measures of plant aerial tissues. Root and shoot growth was more affected by soil water content than photosynthesis-related characteristics under water deficit conditions. With incidences of drought likely to increase, identification of wheat cultivars that are more tolerant of these conditions is important.

## Introduction

Water stress has a negative effect on plant growth and can sharply decrease plant productivity (Pan *et al*. 2002). Roots play a key role in water and nutrient supply for plants. However, the physiochemical and biological status of the surrounding soil can have a significant effect on their activity, particularly the relationship between root development and soil pore structure, which is both complex and relatively unexplored (Carminati *et al.* 2010; Mooney *et al.* 2012).

Roots are morphologically and functionally structured for water, mineral and nutrient absorption from the soil by apoplastic and/or symplastic traits. Reduction in plant output is mainly observed when plants are exposed to water deficiency during long periods, which affects almost all physiological processes, involving growth (McDonald and Davies 1996), stomatal conductance and photosynthesis (Flexas *et al.* 2004). During drought (i.e. substantial and sustained reduction in soil water availability), plant aerial tissues (leaves and stems) can be significantly inhibited in growth, while the roots continuously grow to explore the soil volume for water and nutrients (Sharp and Davies 1989). The regulation of shoot–root interactions has been a subject of intense study but many aspects remain unexplored partly due to the difficulty of studying the dynamics of complex root systems when embedded in soil.

Water shortage is a global problem affecting the development of agricultural crops and the maintenance of food production (Jaleel *et al.* 2007). Increases or decreases in temperature and water availability specifically affect wheat productivity and photosynthetic efficiency (Wang *et al.* 2008). In wheat, grain yield is the function of the number of plants per ha, the number of fertile tillers per plant, the number of grains per spike and individual grain weight. Yield is affected directly or indirectly by water shortage through these components (Fábián *et al.* 2011). Drought at the last two stages, double ridge to anthesis and anthesis to maturity (GS2 and GS3), is a frequent phenomenon resulting in wheat yield loss (Pradhan *et al.* 2012). However, drought and/or heat stress during early growth stages has been well studied and also impacts on productivity (Madhu and Hatfield 2013; Saeidi *et al.* 2015). Water deficit imposed during the vegetative stage reduces biomass, grain yield and the number of grains per spike.

 Photosynthesis is very sensitive to water deficit and has a direct impact on growth. During the onset of drought, a reduction in stomatal conductance can reduce availability of CO_2_ for photosynthesis and but can, in more severe conditions, be followed by a subsequent inhibition of underlying biochemical processes such as Rubisco carboxylation and electron transport activity, relative water content and even pigment content (Cui *et al.* 2015; Saeidi *et al.* 2015; Flexas *et al.* 2018). It is clear therefore that strategies for enhancing and conserving soil water act to maintain leaf gas exchange and substantially contribute to biomass, the capacity for grain filling and ultimately, the yield of wheat (Xue *et al.* 2006; Lan-Ping *et al.* 2011).

There is a need to determine the effects of water deficit (during periods of vegetative and reproductive growth) on the links between root growth and shoot function. In particular, there is a paucity of knowledge on relationships between 3D root structure, leaf gas exchange traits and growth and whether there remains genetic variation among wheat genotypes for such traits that will enable improved photosynthesis, water use efficiency and drought tolerance (Liu *et al.* 2016)

New developments in imaging technologies, such as X-ray micro-Computed Tomography (μCT), to visualize the roots of plants grown in soil have shown much promise (e.g. Tracy *et al.* 2010; Aravena *et al.* 2011; Schmidt *et al.* 2012). Whilst many studies have examined the impact of water stress on shoot and root growth and photosynthesis, no previous study has visualized the root systems of wheat under drought conditions to measure undisturbed root characteristics in 3D when grown in soil and linked such observations with shoot properties, in particular, photosynthetic gas exchange.This study aimed to investigate the impact of water stress on root and shoot growth in wheat in different soil textural types. We hypothesized that there is a ‘sweet spot’ in soil water content, between 100 and 25 % of field capacity (FC) where wheat root and shoot growth and development parameters are optimized. It was also hypothesized that root and shoot growth under water deficiency would perform better in a clay loam than in a sandy loam soil due to enhanced water retention under stress. Finally, as X-ray μCT imagery was used to visualize root architecture and exposure to X-rays has the potential for negative impacts on plant growth, we assessed both non-scanned and scanned samples hypothesizing that X-ray imaging (using the parameters in this experiment) would not negatively impact on plant growth.

## Materials and Methods

### Sample preparation and X-ray μCT

Soil from a Newport series sandy loam (FAO Class brown soil) and a Worcester series clay loam soil (FAO Class argillic pelosol) was collected from two adjacent fields both under winter wheat at the University of Nottingham experimental farm at Bunny, Nottinghamshire, UK (52.8633°N, 1.1394°W). An independent on-farm survey undertaken at the same time suggested there were no significant differences in the available nutrient status between the soils. The soil was air-dried and sieved to <2 mm before being uniformly packed into columns (15 cm height × 5 cm diameter) in an air-dried state to a representative field bulk density of 1.24 g cm^−3^. Four moisture treatments were chosen to represent a range of saturated, field and dry conditions namely 100, 75, 50 and 25 % of a notional FC, i.e. the soil water content 48 h after drainage from saturation. Four replicates were prepared for each soil type and treatment combination to give a total of 64 columns, of which 32 were μCT scanned at two time intervals (14 and 21 days). An equal number of columns in the treatment structure were prepared but not scanned to assess for any potential effects on the plants by exposure to X-rays during scanning process. After packing, all columns were water-saturated from the base upwards and then drained to reach to different levels of FC (100 %, i.e. 2 days after drainage) and further drained to reach the following levels, after which they were maintained at that level: 75, 50, and 25 % FC. In some cases there was a minor (<1–2 mm) drop in the length of the soil column due to settling however this was variable between treatments thus it was not adjusted for to ensure all columns were treated equally. Seeds of the winter wheat Zebedee (Redigo Deter) were germinated for 48 h before being planted 5 mm below the soil surface. Thereafter, they were placed in a growth room under conditions of 28/22 °C day/night with a 16-h photoperiod at 50 % relative humidity (RH). All columns were placed in a transpired propagator to maintain high RH levels during germination and seedling growth. They were weighed daily and sufficient water was added to maintain soil moisture content at the four predetermined moisture contents. Details of the exact water content, examples of representative matric potentials derived from a water release curve for the same soil texture **[see****Supporting Information—Fig. S1****]** and other relevant soil properties including soil elemental analysis expressed as total concentrations are given in Table 1. The columns were X-ray scanned at Day 14 and 21 after germination using a Phoenix Nanotom® (GE Measurement & Control Solutions, Wunstorf, Germany) μCT scanner set at 110 kV and 130 μA, with a 0.15-mm copper filter and an image averaging of 3. Voxel resolution was set at 55 μm and each scan took 32.5 min to complete. For each column, 1300 image projections were collected on all sampling dates and each image volume had a file size of ~2 GB. Thereafter, these images were reconstructed using datos|x 2.2 software.

**Table 1.** Selected soil physical and chemical properties for the two soil textures. The values of volumetric water content (VWC), matric potential (ψ) (−kPa) at different water content (100, 75, 50 and 25 % FC), the percentage of the sand, silt, the clay, Organic Matter by Loss on Ignition, pH and nutrient content for the sandy loam and the clay loam soils in Newport and Worcester. Note that matric potential values (ψ) were approximated from the water release curve for the specific soil type and not directly measured. Soil nutrients expressed as total concentrations.

Soil properties	Sandy loam	Clay loam
VWC (g g^−1^) at 100 % FC	0.42	0.45
VWC (g g^−1^) at 75 % FC	0.32	0.34
VWC (g g^−1^) at 50 % FC	0.21	0.23
VWC (g g^−1^) at 25 % FC	0.11	0.11
Ψ (−kPa) at 100 % FC	5	30
Ψ (−kPa) at 75 % FC	10	150
Ψ (−kPa) at 50 % FC	90	1200
Ψ (−kPa) at 25 % FC	800	1500
% sand	79	38
% silt	4	31
% clay	17	31
Organic Matter by Loss on Ignition (%)	3.9	4.8
pH	6.9	7.7
Carbon (mg kg^−1^ soil)	17 355	26 768
Nitrogen (mg kg^−1^ soil)	1486	1505
Phosphorus (mg kg^−1^ soil)	778	797
Potassium (mg kg^−1^ soil)	10 384	20 659
Magnesium (mg kg^−1^ soil)	2352	14 150
Sulphur (mg kg^−1^ soil)	2258	2058
Sodium (mg kg^−1^ soil)	1479	3423
Calcium (mg kg^−1^ soil)	2071	13 150

### Image processing and analysis

Root systems were non-destructively segmented from the grey-scale μCT images using the Region Growing selection tool in VG StudioMAX software as described by Tracy *et al.* (2012). The root system models segmented from the μCT image data were used for quantitative determination of total root volume and mean root diameter. Region Growing classifies voxels in a certain grey-value range from a starting seed point. Tolerance values were adjusted to ensure that only root material was included in the growing region of interest from the original seed points. The mean root diameter was measured by the distance measurement tool. As the study was concerned with the interaction between roots and soil, the soil pore characteristics were also measured in addition to root measurements by setting an automatic threshold (the Li algorithm) to segment the pores by selection of the volume of air space for the given spatial resolution (i.e. water-filled pores are not measured by this approach) using ImageJ (Rasband 2018).

### Plant measurements

Photosynthesis (*A*), stomatal conductance (*g*_s_) and transpiration (*T*) were measured the day before and after scanning by infrared gas analysis (LI-6400XT Portable Photosynthesis System, Licor, Lincoln, NE, USA). Measurements took place within the growth room. The settings were as follows: cuvette (block) temperature 30 °C, sample CO_2_ 400 μmol mol^−1^, 50 % (ambient) RH, flow rate 500 μmol s^−1^ and 1500 μmol m^−2^ s^−1^ (saturating) photosynthetically active radiation. Leaves were placed in the cuvette for 2–3 min and allowed to stabilize before measurements were made.

The columns were X-ray scanned during the light part of the photoperiod in randomized order to ensure that all four treatment combinations were equally exposed to any diurnal variation in root growth that may have occurred. After µCT scanning, the columns were dismantled, the roots were washed from the soil and analysed using WinRHIZO®2002c scanning equipment and software to calculate total root volume and mean root diameter (at a 1 pixel = 0.69 mm resolution). The images obtained were collected to compare with the X-ray μCT images. Root and shoot dry weights were also measured by placing in an oven at 75 °C temperature for 24 h.

### Statistical analysis

The results were analysed by general analysis of variance (ANOVA) including water, soil types and their interaction as explanatory variables using Genstat 15.1. All data were first tested for normality and transformed if appropriate. In addition, regression analysis was used to test the relationship between the two root system visualization methods (WinRHIZO and X-ray CT).

## Results

### Root characteristics

#### Total root volume.

**Figure 1.**
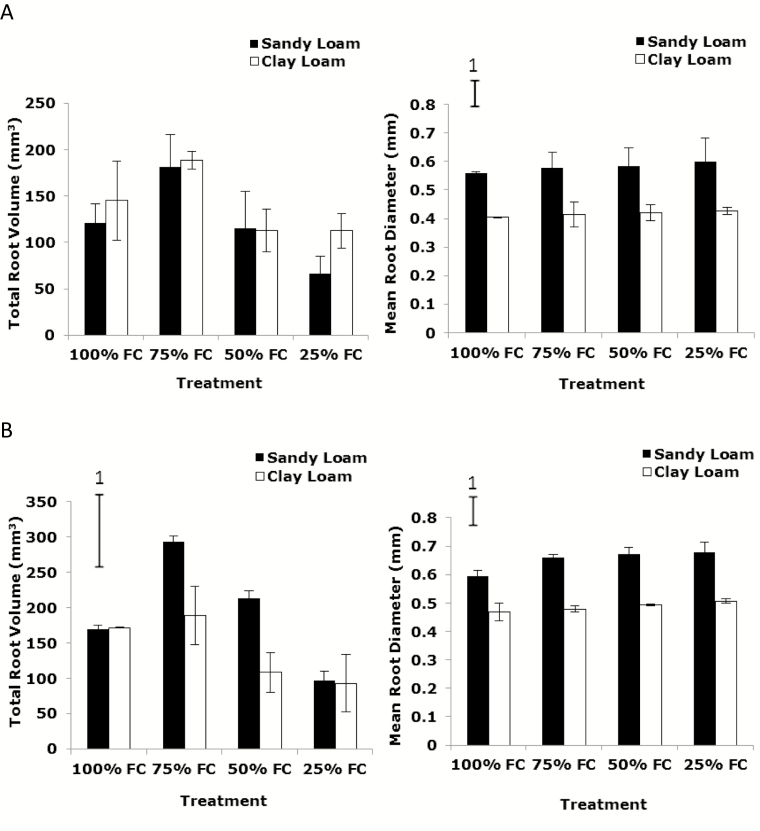
The effect of water stress on total volume and mean root diameter by X-ray CT in wheat variety Zebedee grown in the sandy loam and clay loam soils types 14 (A) and 21 (B) days after germination. Bars indicate means ± SD (*n* = 4). General analysis of variance (ANOVA) showed soil × water interaction on total root volume at 21 days (B) at *P* < 0.01 and mean root diameter at both dates at *P* < 0.05. Vertical bar (1) represents standard errors of difference (SED) between means where interaction is significant.

**Figure 2.**
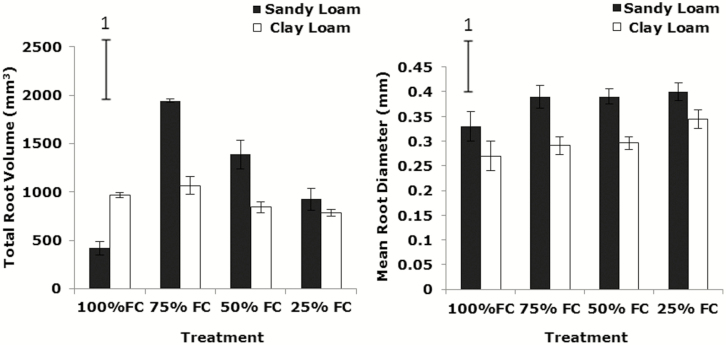
The effect of water stress on total volume and mean diameter of root by WinRHIZO in wheat variety Zebedee grown in the sandy loam and the clay loam soils types 21 days after germination. Bars indicate means ± SD (*n* = 4). General analysis of variance (ANOVA) showed soil × water interaction on total root volume and mean root diameter at *P* < 0.05. Vertical bar (1) represents standard errors of difference (SED) between means where interaction is significant.

 Total root volume by µCT at 14 days post-germination reduced significantly with decreasing soil moisture content (from 75 to 25 % FC) in both soil types (sandy loam and clay loam) (*P*_water_ < 0.01, *P*_soil_ > 0.05, *P*_soil * water_ > 0.05) (Fig. 1A; Table 2). At 21 days post-germination, total root volume by µCT also dropped dramatically with declining soil water content (from 75 to 25 % FC) in the sandy loam and clay loam (*P*_soil_ < 0.01, *P*_water_ < 0.001) (Fig. 1B). The soil * water interaction was significant (*P*_soil * water_ < 0.01). Similar trends were found for WinRHIZO®-derived total root volume which decreased greatly with decreasing soil water content in the sandy loam and clay loam (*P*_water_ < 0.05, *P*_soil * water_ < 0.05) (Fig. 2). Total root volume in the sandy loam was significantly greater than in the clay loam soil (*P*_soil_ < 0.01) (Figs 1B and 2).

**Table 2.** *P*-values by general analysis of variance (ANOVA) for all variables of wheat variety Zebedee grown at different water content (100, 75, 50 and 25 % FC) in the sandy loam and the clay loam soil types 14 (A) and 21 (B) days after germination.

Measurements/treatment	Soil	Water	Soil * water
A			
Total root volume (X-ray)	*P* > 0.05	*P* < 0.01	*P* > 0.05
Mean root diameter (X-ray)	*P* < 0.001	*P* > 0.05	*P* < 0.05
Photosynthesis	*P* < 0.05	*P* > 0.05	*P* > 0.05
Stomatal conductance	*P* < 0.001	*P* < 0.001	*P* > 0.05
Transpiration	*P* < 0.001	*P* < 0.001	*P* < 0.05
inWUE	*P* < 0.001	*P* < 0.05	*P* > 0.05
iWUE	*P* < 0.001	*P* = 0.001	*P* < 0.001
Shoot length	*P* < 0.001	*P* < 0.001	*P* > 0.05
Air-filled porosity	*P* < 0.001	*P* < 0.001	*P* < 0.05
Volumetric water content	*P* < 0.001	*P* < 0.001	*P* < 0.001
Measurements/treatment	Soil	Water	Soil * water
B			
Total root volume (X-ray)	*P* < 0.01	*P* < 0.001	*P* < 0.01
Mean root diameter (X-ray)	*P* < 0.001	*P* < 0.05	*P* < 0.05
Total root volume (WinRHIZO)	*P* < 0.01	*P* < 0.05	*P* < 0.05
Mean root diameter (WinRHIZO)	*P* < 0.001	*P* < 0.05	*P* < 0.05
Photosynthesis	*P* > 0.05	*P* < 0.001	*P* > 0.05
Stomatal conductance	*P* < 0.01	*P* < 0.001	*P* > 0.05
Transpiration	*P* > 0.05	*P* < 0.001	*P* > 0.05
inWUE	*P* < 0.05	*P* > 0.05	*P* < 0.05
iWUE	*P* > 0.05	*P* > 0.05	*P* < 0.01
WUE (shoot dry weight/total water applied)	*P* < 0.001	*P* < 0.001	*P* > 0.05
WUE (root dry weight/total water applied)	*P* < 0.01	*P* < 0.001	*P* > 0.05
WUE (total dry weight/total water applied)	*P* < 0.001	*P* < 0.001	*P* > 0.05
Shoot dry weight	*P* < 0.001	*P* < 0.05	*P* < 0.05
Root dry weight	*P* > 0.05	*P* > 0.05	*P* > 0.05
Shoot length	*P* < 0.001	*P* < 0.001	*P* < 0.001
Total dry weight	*P* < 0.001	*P* < 0.05	*P* > 0.05
Air-filled porosity	*P* < 0.001	*P* < 0.001	*P* > 0.05
Volumetric water content	*P* < 0.01	*P* < 0.001	*P* < 0.001

#### Mean root diameter.

Mean root diameter in the sandy loam soil was significantly greater than in clay loam soil. Mean root diameter by µCT at 14 days post-germination did not significantly increase with decreasing soil water content in the sandy loam and clay loam (*P*_soil_ < 0.001, *P*_water_ > 0.05, *P*_soil * water_ < 0.05) (Fig. 1A). At 21 days post-germination, mean root diameter by µCT increased significantly in the sandy loam and clay loam (*P*_soil_ < 0.001, *P*_water_ < 0.05, *P*_soil * water_ < 0.05) (Fig. 1B). Identical trends were found by WinRHIZO® analysis with mean root diameter increasing significantly with decreasing soil water content in the sandy loam and clay loam (*P*_soil_ < 0.001, *P*_water_ < 0.05, *P*_soil * water_ < 0.05) (Fig. 2). Mean root diameter in the sandy loam soil was significantly greater than in the clay loam (*P*_soil_ < 0.001) (Figs 1 and 2).

### Photosynthesis, stomatal conductance and transpiration

**Figure 3.**
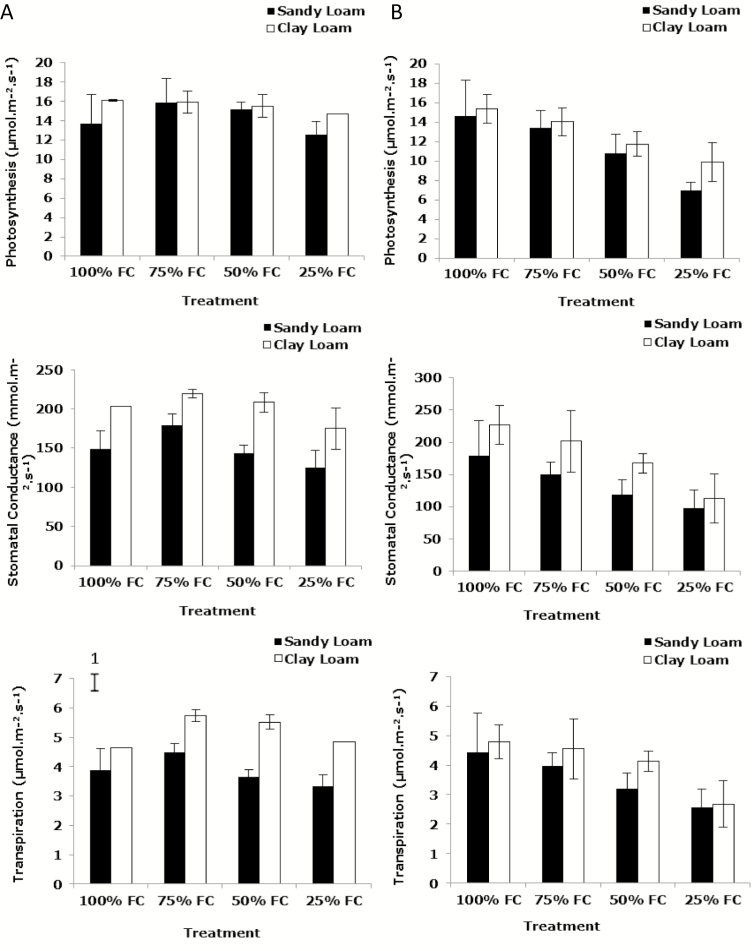
The effect of water stress on photosynthesis (*A*), stomatal conductance (*g*_s_) and transpiration (*T*) of wheat variety Zebedee 14 (column A) and 21 (column B) days post-germination and after scanning in the sandy loam and the clay loam soils types. Bars indicate means ± SD (*n* = 4). General analysis of variance (ANOVA) showed soil × water interaction on photosynthesis, stomatal conductance and transpiration at *P* < 0.05 at 14 days in (column A). Vertical bar (1) represents standard errors of difference (SED) between means where interaction is significant.

At 14 days post-germination, light-saturated photosynthesis (*A*) showed numerically small differences between the soil types; however, it was consistently greater in clay loam than in the sandy loam and showed a significant difference at 100 and 25 % FC (*P*_soil_ < 0.05, *P*_soil * water_ > 0.05) (Fig. 3A). At 14 days there were no significant differences in photosynthesis with respect to soil water content in both the sandy loam and clay loam (*P*_water_ > 0.05). However, at 21 days the pattern was clear with a large progressive, significant decline between 100 and 25 % FC (*P*_water_ < 0.001, *P*_soil_ > 0.05, *P*_soil * water_ > 0.05) (Fig. 3B). Trends in changes in *A* were similar to those of stomatal conductance (*g*_s_) but the magnitude of change was greater in the latter resulting in significant differences. At 14 days after germination *g*_s_ reduced significantly with decreasing soil water content in both soil types (*P*_water_ < 0.001). Notably, stomatal conductance in the clay loam was significantly greater than in the sandy loam at 14 days (*P*_soil_ < 0.001, *P*_soil * water_ > 0.05) and 21 days (*P*_soil_ < 0.01, *P*_soil * water_ > 0.05) (Fig. 3A and B). The effect of the water stress at 21 days after germination on *g*_s_ was similar to that at 14 days post-germination. Transpiration (*T*) changes largely followed those of *g*_s_ (Fig. 3A and B). These data indicate that the imposition of water stress resulted in partial closure of stomata, particularly at 21 days, and consequentially leaf conductance and photosynthetic carbon gain were reduced.

### Intrinsic and instantaneous water use efficiency

**Figure 4.**
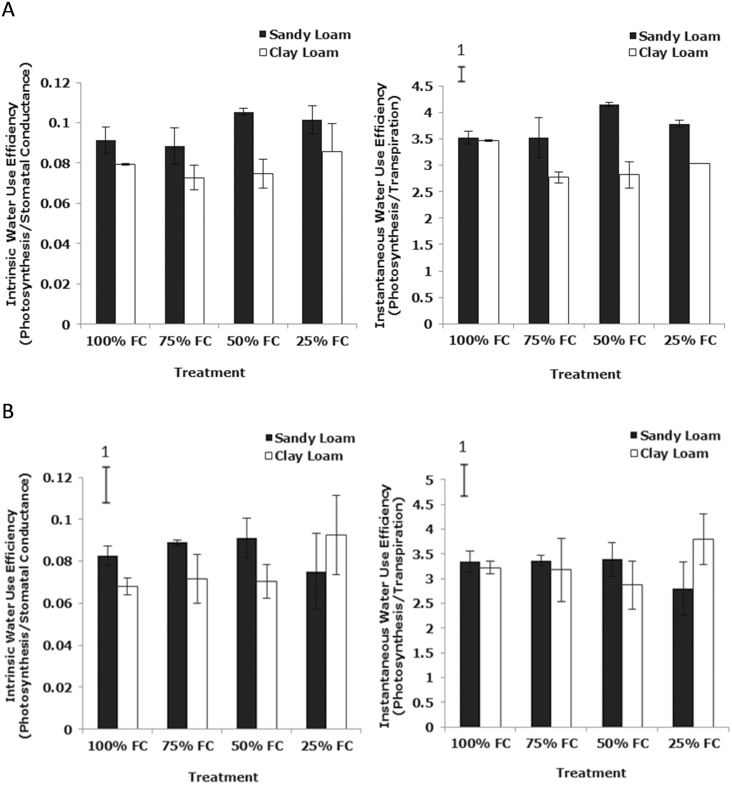
Intrinsic WUE and iWUE of wheat variety Zebedee 14 (A) and 21 (B) days post-germination and after scanning grown under different moisture content (100, 75, 50 and 25 % FC) in the sandy loam and the clay loam soils types. Bars indicate means ± SD (*n* = 4). General analysis of variance (ANOVA) showed soil × water interaction on inWUE at *P* < 0.05 in (B) and iWUE at *P* < 0.001 in (A) and at *P* < 0.01 in (B). Vertical bar (1) represents standard errors of difference (SED) between means where interaction is significant.

To investigate the impact of different levels of water stress on leaf water use efficiency of wheat plants at 14 and 21 days after germination, leaf intrinsic water use efficiency (inWUE) and instantaneous WUE (iWUE) was calculated as *A*/*g*_s_ and *A*/*T*, respectively. Notably, the inWUE 14 days after germination in the sandy loam was significantly greater than in the clay loam (*P*_soil_ < 0.001, *P*_soil * water_ > 0.05) largely due to the lower *g*_s_ in the former. Similarly, inWUE at 21 days after germination in the sandy loam was significantly greater than in the clay loam, except at 25 % FC was lower (*P*_soil_ < 0.05, *P*_soil * water_ < 0.05) (Fig. 4B). The trends for inWUE according to soil water content were similar between time points but varied according to soil type. In the sandy loam it was lower at 100 and 75 % FC compared to 50 and 25 % FC. Clay loam showed a different pattern, declining between 100 % and 75 % and 50 %, though at the 14-day time point it increased at 25 % (*P*_water_ < 0.05) (Fig. 4A). Instantaneous WUE at 14 days post-germination increased with decreasing soil water content in the sandy loam from 100 to 50 % FC, then it decreased to 25 % FC. In the clay loam, it reduced significantly with reducing soil water content from 100 to 75 % FC, then it increased to 25 % FC (*P*_water_ = 0.001). Instantaneous WUE in the sandy loam was significantly greater than in the clay loam (*P*_soil_ < 0.001, *P*_soil * water_ < 0.001) (Fig. 4A). The soil * water interaction was significant for iWUE 21 days post-germination (*P*_soil * water_ < 0.01). There was no significant difference between both soil types and water stress levels (*P*_water_ > 0.05, *P*_soil_ > 0.05) (Fig. 4B).

### Shoot and root mass

**Figure 5.**
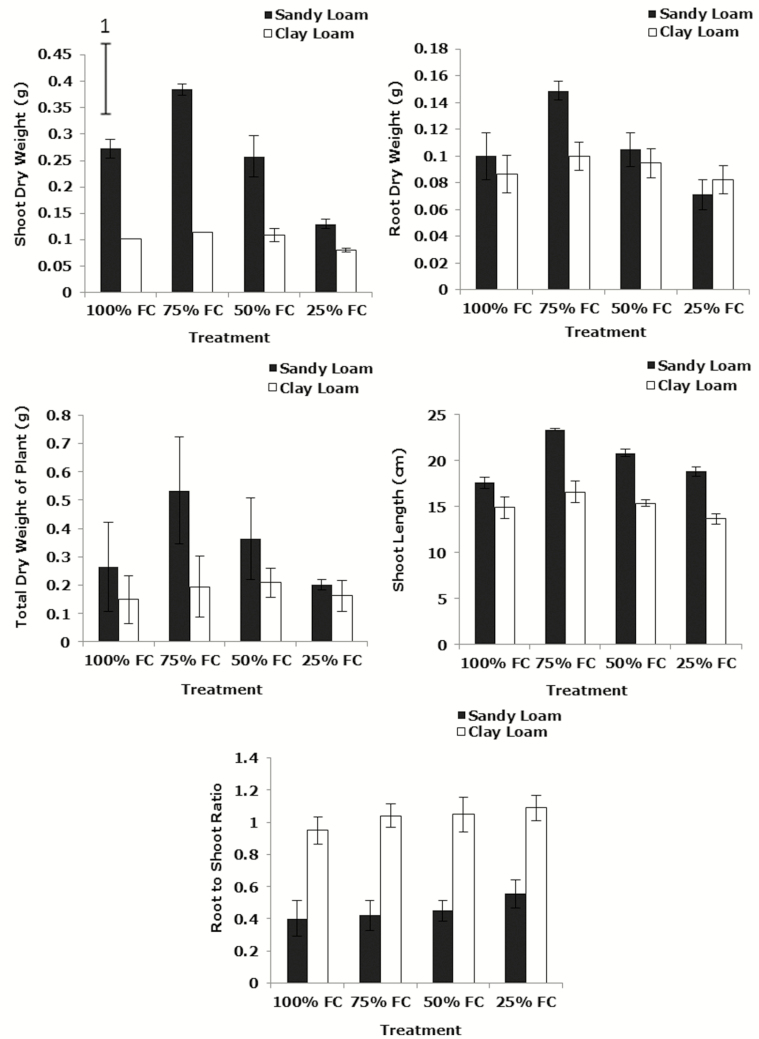
The effect water stress on shoot, root and total dry weight, shoot length and root to shoot ratio (dry weight) of wheat variety Zebedee 21 days post-germination grown under different moisture content (100, 75, 50 and 25 % FC) in the sandy loam and the clay loam soil. Bars indicate means ± SD (*n* = 4). General analysis of variance (ANOVA) showed soil × water interaction on shoot, root and total dry weight, shoot length and root to shoot ratio (dry weight) at *P* < 0.05. Vertical bar (1) represents standard errors of difference (SED) between means where interaction is significant.

Measurements of dry weight and gross morphology indicated substantial differences in the influence of soil type. Shoot dry weight in the sandy loam was significantly greater than in clay loam (*P*_soil_ < 0.001, *P*_soil * water_ < 0.05) (Fig. 5). Total dry weight in sandy loam was also significantly greater than in clay loam (*P*_soil_ < 0.001, *P*_soil * water_ > 0.05) and the same applied to shoot length (*P*_soil_ < 0.001, *P*_soil * water_ > 0.05). There were fewer differences in root dry weight but there was a clear difference in the investment into roots, root to shoot ratio at 21 days post-germination in the clay loam was significantly greater (*P*_soil_ < 0.001, *P*_water_ > 0.05, *P*_soil * water_ > 0.05) (Fig. 5). These differences are consistent with the higher water use efficiency in sandy soil but not with the measurements of *A*. Examples of images of Zebedee wheat leaves under different soil water content (100, 75, 50 and 25 % FC) in the sandy loam and the clay loam soils at 21 days after germination are shown in **Supporting Information—Fig. S2**.

The influence of soil water was more pronounced in the sandy loam soil than clay loam (Fig. 5). Shoot dry weight at 21 days post-germination decreased significantly with reducing soil water content in sandy loam, while it decreased only slightly in the clay loam (*P*_water_ < 0.05). Root dry weight decreased greatly with reducing soil water content in the sandy loam, while it decreased slightly in the clay loam from 75 to 25 % FC (*P*_water_ > 0.05, *P*_soil_ > 0.05, *P*_soil * water_ > 0.05). Total dry weight decreased significantly with decreasing soil water content in the sandy loam, while it increased slightly in the clay loam from 100 to 50 % FC, then it decreased to 25 % FC (*P*_water_ < 0.05). Shoot length at 14 days after germination in both soil types declined significantly with decreasing soil water content (*P*_water_ < 0.001). Shoot length at 21 days post-germination in the sandy loam was significantly greater than in the clay loam (*P*_soil_ < 0.001, *P*_soil * water_ < 0.001) and also decreased significantly with decreasing soil water content in both soil types (*P*_water_ < 0.001).

### Whole-plant water use efficiency

**Figure 6.**
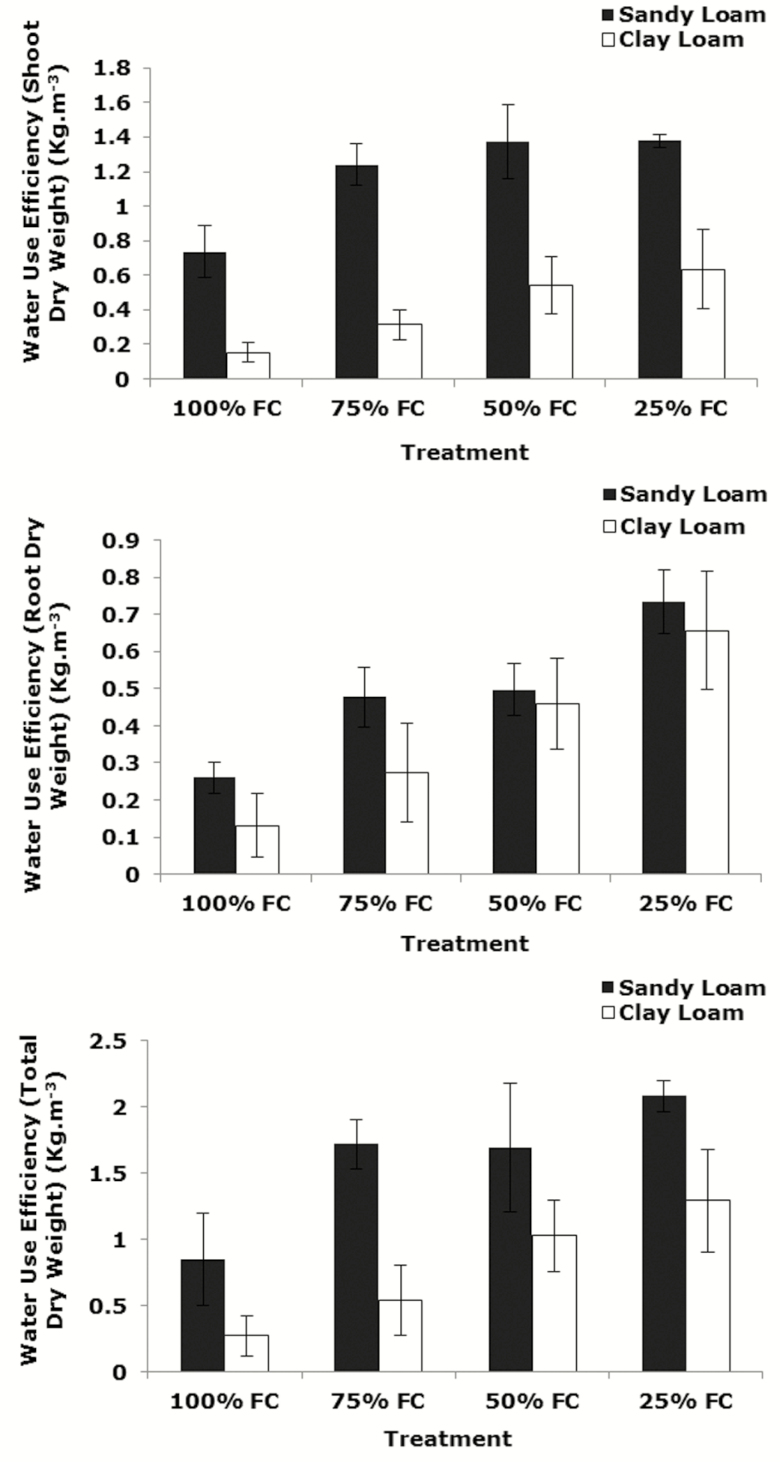
Water use efficiency (based on shoot, root and total dry weight) of wheat variety Zebedee grown under different moisture content (100, 75, 50 and 25 % FC) 21 days after germination in the sandy loam and the clay loam soils. Bars indicate means ± SD (*n* = 4). General analysis of variance (ANOVA) showed soil × water interaction on water use efficiency.

Water use efficiency (based on shoot and root dry weight) increased significantly with decreasing soil water content in both soil types (*P*_water_ < 0.001, *P*_soil * water_ > 0.05). Water use efficiency in the sandy loam was significantly greater than in the clay loam (*P*_soil_ < 0.001, *P*_soil_ < 0.01 for water use efficiency (based on shoot and root dry weight, respectively)) (Fig. 6) which demonstrates some similarities with the leaf gas exchange values. Water use efficiency based on total dry weight at 21 days post-germination increased significantly with decreasing soil water content in both soil types. It increased in the sandy loam from 100 to 75 % FC, then it slightly reduced to 50 % FC, then it increased to 25 % FC. Similarly, in the clay loam, it increased significantly from 100 to 25 % FC (*P*_water_ < 0.001). Water use efficiency in the sandy loam was significantly greater than in the clay loam (*P*_soil_ < 0.001, *P*_soil * water_ > 0.05) (Fig. 6).

### Soil physical properties

Air-filled porosity (as determined by X-ray imagery at a resolution of 55 µm) at 14 and 21 days post-germination increased significantly with decreasing soil water content in both soil types (*P*_water_ < 0.001) **[see****Supporting Information—Fig. S3****]**. Air-filled porosity at this scale in the sandy loam was significantly greater than in the clay loam soil (*P*_soil_ < 0.001, *P*_soil * water_ < 0.05) at 14 and 21 days (*P*_soil_ < 0.001, *P*_soil * water_ > 0.05) **[see****Supporting Information—Fig. S3****]**. Volumetric water content (by weight) in the clay loam was significantly greater than in the sandy loam soil at all levels of water stress in both soil types, except at 100 % FC when it was lower at 14 days (*P*_soil_ < 0.001) and at 21 days (*P*_soil_ < 0.01) post-germination. The soil * water interaction was significant for volumetric water content (*P*_soil * water_ < 0.001) **[see****Supporting Information—Fig. S3****]**.

### Effect of X-ray exposure on plant growth

As the use of µCT is rapidly expanding in studies of this nature, we considered it important to investigate the potential effect of X-ray exposure on root traits; therefore, WinRHIZO-derived root data were compared between scanned and non-scanned plants. No significant nor noticeable effect of X-ray exposure was observed on wheat plants following scanning (*P* > 0.05). Scanned root systems had an average total root volume of 991 cm^3^ compared to 985 cm^3^ for non-scanned plants (*P* > 0.05). Root diameter was c. 0.36 mm for both scanned and non-scanned plants, respectively (*P* > 0.05) **[see****Supporting Information—Fig. S4****]**. Photosynthesis parameters were measured on plants before and after scanning and no significant nor observable effect of X-ray exposure on photosynthesis parameters was associated with scanning (*P* > 0.05). Photosynthesis was 11.95 and 12.12 µmol m^−2^ s^−1^ before and after scanning (*P* > 0.05). Stomatal conductance averaged 157.2 and 162.1 mmol m^−2^ s^−1^ before and after scanning plants, respectively (*P* > 0.05). Transpiration of plants before and after scanning also averaged 3.7 and 3.8 µmol m^−2^ s^−1^**[see****Supporting Information—Fig. S5****]**.

## Discussion

**Figure 7.**
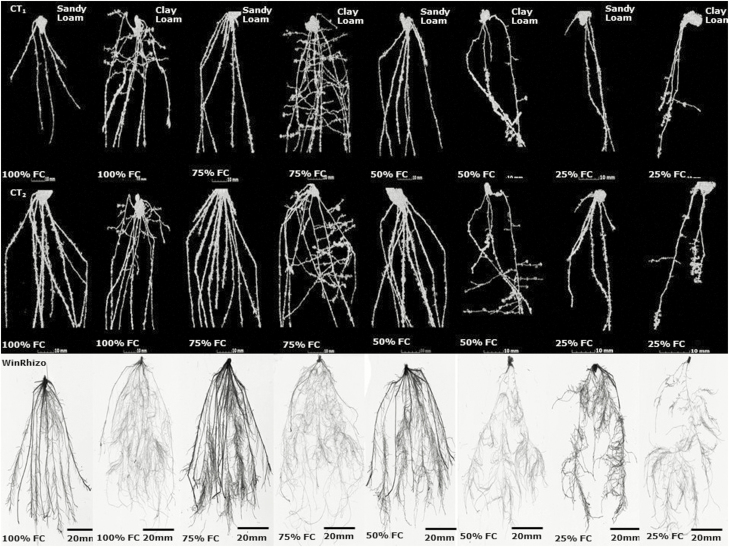
CT images of the roots of Zebedee 14 (CT_1_) and 21 (CT_2_) days after germination grown under water deficit (100, 75, 50 and 25 % FC) in the sandy loam and the clay loam soils and root systems (at 21 days) of the same plants after extraction and analysis using WinRHIZO® equipment compared to CT_2_ images. Scale bars represent 10 and 20 mm.

Drought is an important environmental factor limiting crop growth and yield. We found severe water stress (at 25 % FC) significantly negatively affected total root volume in both soils (sandy loam and clay loam) as hypothesized. There was also a significant soil * water interaction for total root volume probably due to the differential responses to water stress, as shown by root architecture (Fig. 7). Soil texture is highly influential for root architecture, impacting the mechanical impedance (physical stress) on root elongation through the soil, as well as affecting the availabilities of water, oxygen and nutrients (Gregory 2006; Helliwell *et al.* 2019). We hypothesised Zebedee would grow better in clay loam rather than sandy loam soil due to higher water availability. However, we observed the converse, which could be due to the formation of cracks in clay soil (Whitmore and Whalley 2009) promoting evaporation although we saw no clear evidence of this in the CT images. Another influencing factor might be increased soil hardness and strength (Whalley *et al.* 2006, 2008) associated with soil drying. As soil strength increases, root elongation rate decreases due to increased resistance of soil particles to displacement. Strong soil is a serious problem as it can restrict access of roots, typically at depth to water and nutrients (Clark *et al.* 2003) and decreases plants growth. It is important to note the soils in this study were not field structured, but repacked and as a result, the clay loam soil sieved to <2 mm contained a larger portion of macropores than likely to exist in the field, which would have contributed to increased drainage and reduced water retention.

Mean root diameter in the sandy loam soil was thicker than in the clay loam which is most likely due to the soil strength/bulk density of the soil. Although the soils were originally packed the same bulk density (1.24 g cm^−3^), this may have changed over the course of the experiment in response to the water treatment and root development. Thicker root diameters in the sandy soils over clay loam soils have been reported previously (Tracy *et al.* 2013). Mean root diameter at 100 % FC increased sharply from 100 to 75 % FC, while it was slightly increased from 75 to 25 % FC which is more likely due to water stress. Aggarwal *et al.* (2006) also found greater root diameters in drier soil conditions, whereas Muñoz-Romero *et al.* (2010) suggested root diameter can decrease during dry conditions. Smaller root diameters in drier conditions can occur due to greater resistance to penetration (Muñoz-Romero *et al.* 2010), which is important because Clark *et al.* (2008) showed good root penetration was consistently associated with greater root diameter. Thick roots have a greater capability for water absorption from deeper soil layers (Yambao *et al.* 1992) and confer greater water deficiency tolerance through root branching (which is associated with root thickness) (Fitter 1991). Thick roots persevere for longer periods and produce more and larger root branches, therefore increasing water absorption capacity (Ingram *et al.* 1994).

The number of main roots decreased significantly with reducing soil water content (Fig. 7). Water deficiency has a negative effect on plant growth and development and sharply decreases plant productivity (Pan *et al.* 2002). Gao *et al.* (2007) also showed water deficit hinders total root and shoot function and decreases root–shoot interactions. A more serious water deficit leads to a longer period of unstable growth for a plant. Rewatering can subsequently enhance root–shoot interactions, increase dry matter accumulation rate and prolong the duration of instable growth (Gao *et al.* 2007). Root to shoot ratio increased with worsening water deficit and was significantly greater in the clay loam rather than sandy soil. This suggests dry climate genotypes (where sandy soils are often more prevalent) might have stronger drought resistance through a well-developed root system (Wu and Bao 2011). Longer roots have been shown to be very important for crop yield under water stress conditions (Ali *et al.* 2009) though Khan *et al.* (2010) found shoot growth was more affected than root length by water stress. The reason for increased root to shoot ratio could be due to limited supply of water and nutrients; hence, root growth occurs at the expense of shoot (Khan *et al.* 2010).

The notional FC (100 % FC) used in this study had a negative impact on root and shoot growth mostly likely due to excessive soil moisture. High soil water content leads to hypoxia as oxygen is needed in the soil for roots to respire (Yavas *et al.* 2012). Shoot and root characteristics values suggest 75 % FC was optimal in comparison to other water stress levels (100, 50 and 25 %). As FC is affected by so many factors, it cannot be considered as a constant; hence, this result is not surprising.

Drought is a significant environmental restriction which limits the ability of crop photosynthesis to contribute to biomass, and ultimately, yield (Shao *et al.* 2005; Huang *et al.* 2009; Zheng *et al.* 2010). Therefore, we extend the functional implications of root architecture by relating it to shoot gas exchange characteristics. Photosynthesis (*A*), stomatal conductance (*g*_s_) and transpiration (*T*) are highly dependent on root supply of water and nutrients and here were significantly and negatively affected by water stress conditions. Moreover, we noted higher values of *g*_s_ in clay loam versus sandy loam soil, which is important to acknowledge as most previous studies on the effect of water stress on wheat growth have been performed on a single soil type/texture. Higher values of *A*, *g*_s_ and *T* in the clay loam soil indicate more light per unit leaf area could be utilized for carbohydrate production under these conditions of water stress (Wu and Bao 2011), although we note the effect on *g*_s_ and *T* was substantially greater than that of *A* and most pronounced at lower soil water contents.

Photosynthetic efficiency can be affected substantially by water availability (Wang *et al.* 2008) which is typically a complex response to soil water, atmospheric humidity and temperature. *A* versus *g*_s_ responses are not fixed and it is common to observe a reduction in *g*_s_ before any severe retardation of *A* occurs resulting in an increase in iWUE (e.g. Caine *et al.* 2019). We see this in terms of the differences between soil types and water availability and it can be considered as a first stage in drought progression when mild water deficiency occurs beyond which stomata will limit leaf conductance and gas diffusion to an extent that severely limits photosynthetic rate. It is also possible the transpiration rate here may have contributed to some partial depletion of soil water in between regulated watering events. Other non-stomatal effects can limit photosynthesis exist at severe water stress such as Rubisco deactivation and photoinhibition (Santos *et al.* 2009; Yu *et al.* 2009; Souza *et al.* 2010). It is unclear whether the latter effects would be present here.

The observation of a lower *g*_s_ which does not, under these conditions, affect *A* is quite important in terms of crop water use efficiency and drought tolerance. For example, recent work has shown that restricting *g*_s_ in cereal species by lowering stomatal density helps to conserve soil water and maintain long-term photosynthesis during periods of soil water deficit (Hughes *et al.* 2017; Mohammed *et al.* 2019).

By inference from the soil water release curve **[see****Supporting Information—Fig. S1****]**, we propose the optimal matric potential for root and shoot growth was ~−10 kPa (0.32 g g^−1^) in the sandy loam and −150 kPa (0.34 g g^−1^) in the clay loam soil at 75 % FC. However, this differs for photosynthesis, stomatal conductance and transpiration; −5 kPa (0.42 g g^−1^) in the sandy loam and −30 kPa (0.45 g g^−1^) in the clay loam soil at 100 % FC (Table 1). This suggests root and shoot growth is more affected by soil water content than photosynthesis-related characteristics during water deficit conditions. However, the difference in photosynthesis was not significant between 100 and 75 % FC and only significant between 100 and 50 % FC for clay loam. This disconnect might arise from the fact that the photosynthesis measurements were made at the leaf level but that growth rate results from whole-plant gas exchange. Leaf area was higher for the sandy loam plants **[see****Supporting Information—Fig. S2****]** meaning that the plants would intercept more light and could attain a higher growth rate with the same or even lower photosynthetic rate per unit leaf area. Other physiological processes might be important such as low rate of respiration which might have hindered the plant growth. However, Batool *et al.* (2015) reported *Abelmoschus esculentus* had a higher rate of photosynthesis at mild water stress level (60 % FC), but the highest biomass was recorded at low water stress level (100 % FC). This might be due to the high respiration rate which hindered biomass accumulation though differences might be plant-specific. In contrast, Akhkha *et al.* (2011) found water deficit affected the photosynthesis rates in different wheat cultivars, but to varying extents. Their study indicated moderate water stress (50 % FC) did not affect drought-tolerant wheat cultivars, and the impact on photosynthesis efficiency was most observable in drought-sensitive cultivars. Further, differences in soil nutrient status between the contrasting soil textures cannot be discounted as also exerting some influence on plant growth.

In general, µCT offers an advantage in 3D visualization of the root system architecture (RSA) in addition to the ability to measure the associated soil pore characteristics. However, the disadvantage we observed here is that considerably less of the lateral roots in comparison to the WinRHIZO images are revealed (Fig. 7). Whilst it is possible to undertake imaging at a higher resolution than used in this study and thus potentially visualize more roots, this usually results in the compromise of having to examine a smaller sample size and most likely a shorter growth period (e.g. Tracy *et al.* 2012). In contrast, data obtained from WINRHIZO typically provides more information on the fine roots although some (i.e. an unquantifiable amount) of these are lost in the soil removal process, in addition no geometrical information concerning how the roots and soil interact is given, nor structural information regarding the soil. However, such advantages and disadvantages might be specific to plant type as Galdos *et al.* (2020) recently demonstrated that X-ray CT proved more effective in visualizing the fine roots in selected forage grasses compared with WinRHIZO.

Despite the disadvantages of CT imagery for studies of this kind, there has been a surge in related publications in recent years (e.g. Keyes *et al.* 2013; Burr-Hersey *et al.* 2017; Flavel *et al.* 2017; Helliwell *et al.* 2019). An interesting aspect of the application of µCT in such studies is the ability to visualize root growth in soil over time via repeated scanning such as in Tracy *et al.* (2013); however, this increases the possibility of damage to the plant via the exposure to radiation (Zappala *et al.* 2013). In this study, the plants were only subjected to two scanning events and the scan times were kept as short as possible to achieve the optimum image quality (c. 30 min). No evidence of any damage to the plants from exposure to X-rays was observed. However, as future experiments are likely to consider multiple and longer scans due to larger sample sizes, the assessment for potential X-ray damage compromising experiments should be undertaken routinely.

## Conclusions

Total root volume for the wheat cv. Zebedee decreased significantly due to water stress while the mean root diameter increased significantly. Plant function, assessed by photosynthesis, stomatal conductance and transpiration, decreased significantly with decreasing soil water content. Contrasting results were observed depending on soil texture with photosynthesis in plants grown in a clay loam soil significantly greater than in sandy loam soil. Our notional 100 % FC in this experiment was most almost certainly too wet for optimal growth conditions leading to anoxia. However, the impact of water stress on the precise RSA is not well known, and in this study the root architecture in sandy soil was more developed in terms of lateral root formation than in clay loam which was the converse to our hypothesis. As we recorded no negative effects to repeated X-ray imaging of wheat, we propose our approach should be considered for further experiments to examine the response of different drought-resistant wheat varieties to contrasting water stress conditions though non-scanned samples should retained as a precaution against possible radiation damage.

## Supporting Information

The following additional information is available in the online version of this article—

**Figure S1**. Water release curve for the sandy loam and clay loam soils fitted to the Van Genuchten-Mualem model.

**Figure S2**. Leaves of wheat under different moisture content (100, 75, 50 and 25 % FC) in the sandy loam and the clay loam soils 21 days after germination. Scale bar represents 20 mm.

**Figure S3**. Volumetric water content 14 (A) and 21 (B) and air filled porosity 14 (C) and 21 (D) days post germination in the sandy loam and the clay loam soils. Bars indicate means ± SD (*n* = 4). General analysis of variance (ANOVA) showed soil x water interaction on volumetric water content at both dates. Vertical bar (1) represents standard errors of difference (SED) between means where interaction is significant at *P* < 0.001.

**Figure S4**. Impact of X-ray CT on root growth in wheat variety Zebedee. Zebedee at 21 days was grown under four levels of water stress (100, 75, 50 and 25 % FC) in sandy loam and clay loam soil types. Bars indicate means ± SD (*n* = 4).

**Figure S5**. Impact of X-ray CT on shoot growth in wheat variety Zebedee. Zebedee at 21 days was grown under four levels of water stress (100, 75, 50 and 25 % FC) in sandy loam and clay loam soil types. Bars indicate means ± SD (*n* = 4).

plaa036_suppl_Supplementary_Figures

## Data Availability

Data are available from the following link: doi:10.17639/nott.7063.
