# Supplementary material for: Quantifying the influence of water deficit on root and shoot growth in wheat using X-ray Computed Tomography
Source: AoB Plants. 2020 Jul 26;12(5):plaa036. doi: 10.1093/aobpla/plaa036 (PMC7469715; doi:10.1093/aobpla/plaa036)
Supplement: plaa036_suppl_Supplementary_Figures [file plaa036_suppl_supplementary_figures.docx]

**List of Supplementary Figures**

Supplementary Figure 1: Water release curve for the sandy loam and clay loam soils fitted to the Van Genuchten-Mualem model

Supplementary Figure 2: Leaves of wheat under different moisture content (100%, 75%, 50% and 25% FC) in the sandy loam and the clay loam soils 21 days after germination. Scale bar represents 20mm.

Supplementary Figure 3: Volumetric water content 14 (A) and 21 (B) and air filled porosity 14 (C) and 21 (D) days post germination in the sandy loam and the clay loam soils. Bars indicate means ± S.D (n=4). General analysis of variance (ANOVA) showed soil x water interaction on volumetric water content at both dates. Vertical bar (1) represents standard errors of difference (SED) between means where interaction is significant at P< 0.001.

**Supplementary Figure 4:** Impact of X-ray CT on root growth in wheat variety Zebedee. Zebedee at 21 days was grown under four levels of water stress (100%, 75%, 50% and 25% FC) in sandy loam and clay loam soil types. Bars indicate means ± S.D (n=4).

**Supplementary Figure 5:** Impact of X-ray CT on shoot growth in wheat variety Zebedee. Zebedee at 21 days was grown under four levels of water stress (100%, 75%, 50% and 25% FC) in sandy loam and clay loam soil types. Bars indicate means ±S.D (n=4).


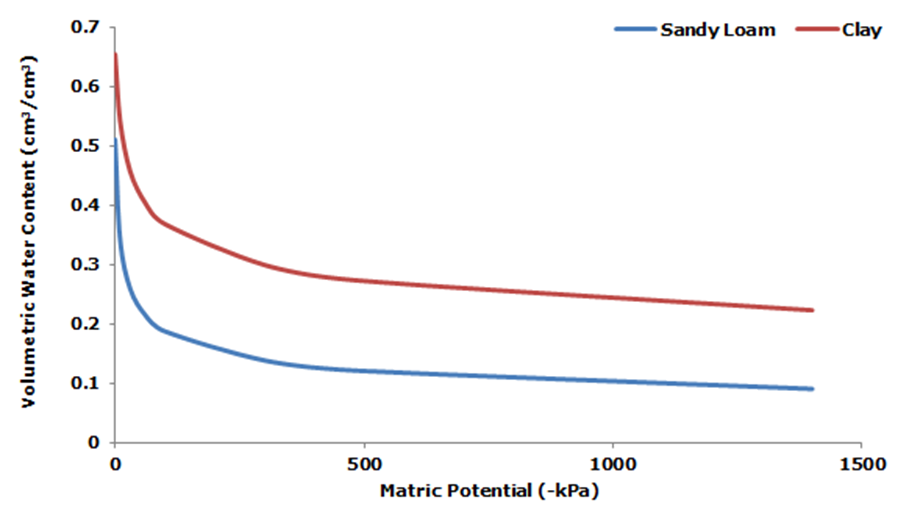


Supplementary Figure 1: Water release curve for the sandy loam and clay loam soils fitted to the Van Genuchten-Mualem model.


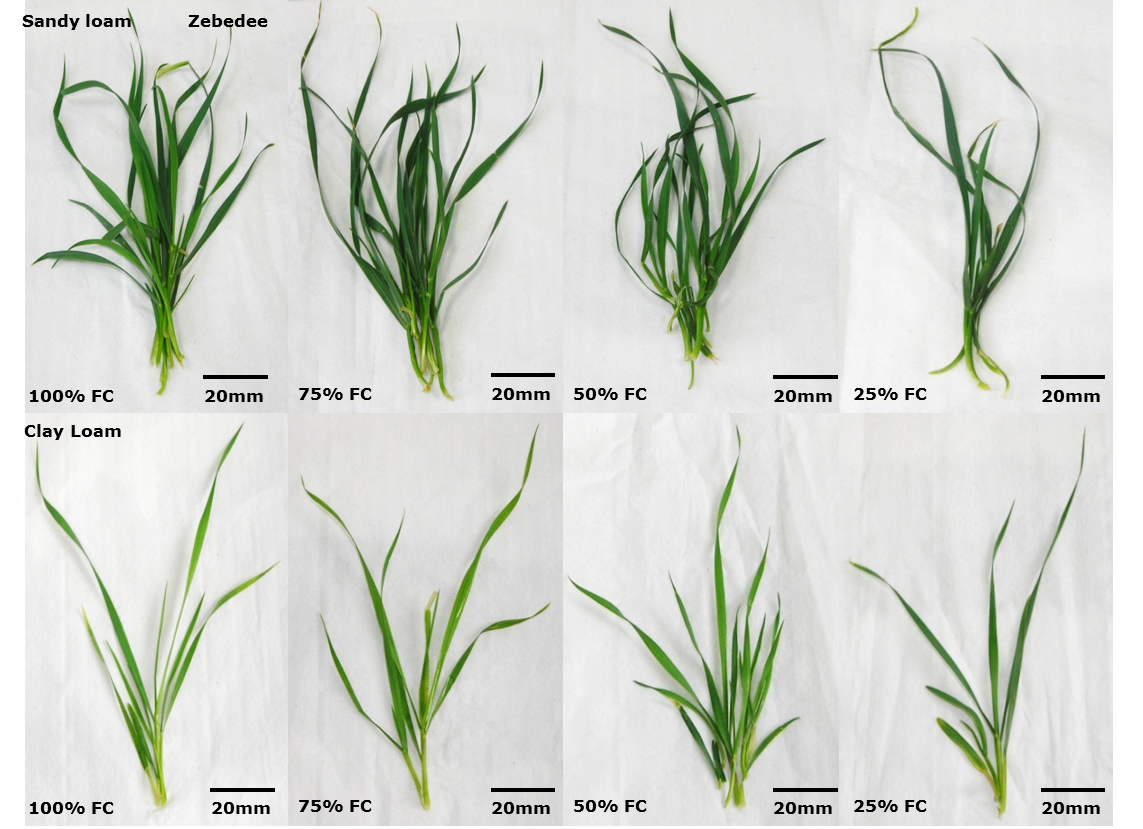


Supplementary Figure 2: Leaves of wheat under different moisture content (100%, 75%, 50% and 25% FC) in the sandy loam and the clay loam soils 21 days after germination. Scale bar represents 20mm.


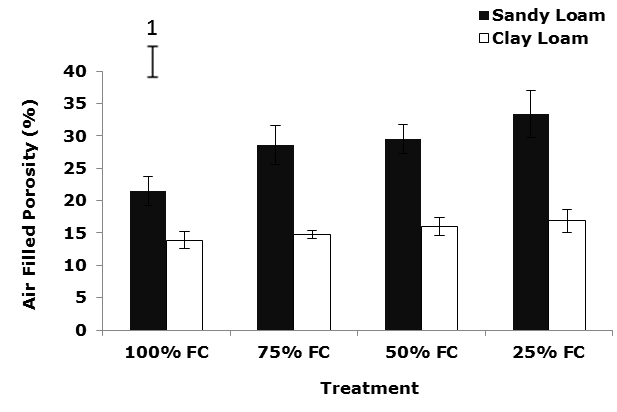

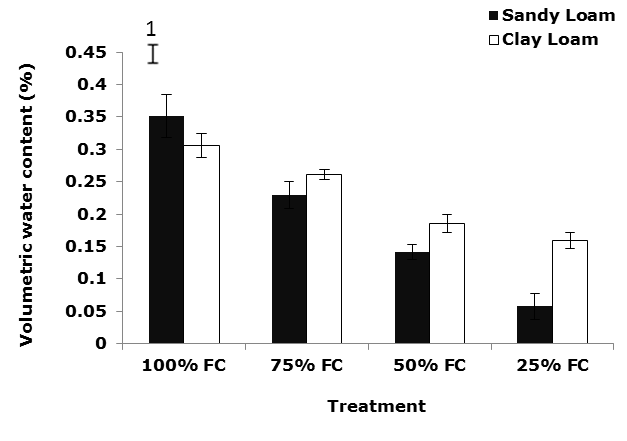
A C


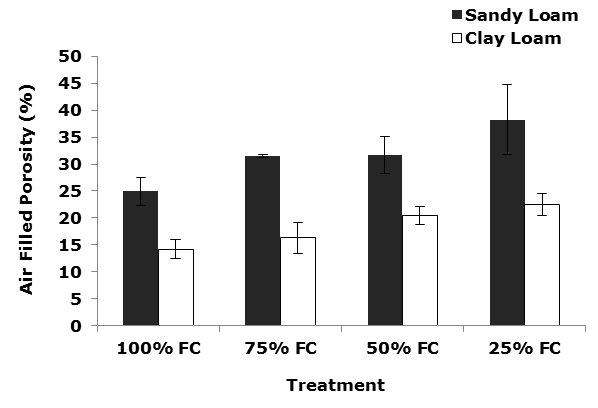


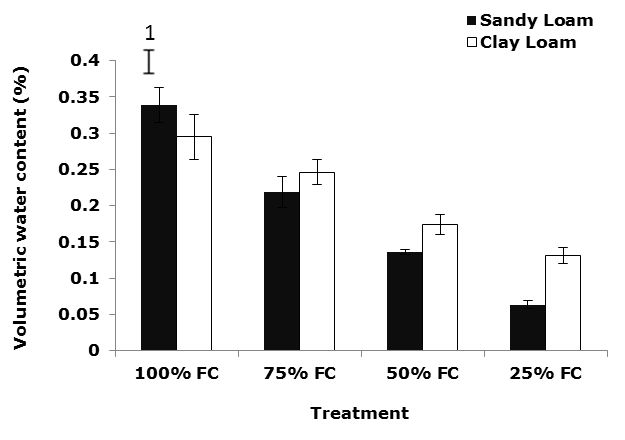
B D

Supplementary Figure 3: Volumetric water content 14 (A) and 21 (B) days post germination in the sandy loam and the clay loam soils. Bars indicate means ± S.D (n=4). General analysis of variance (ANOVA) showed soil x water interaction on volumetric water content at both dates. Vertical bar (1) represents standard errors of difference (SED) between means where interaction is significant at P< 0.001.


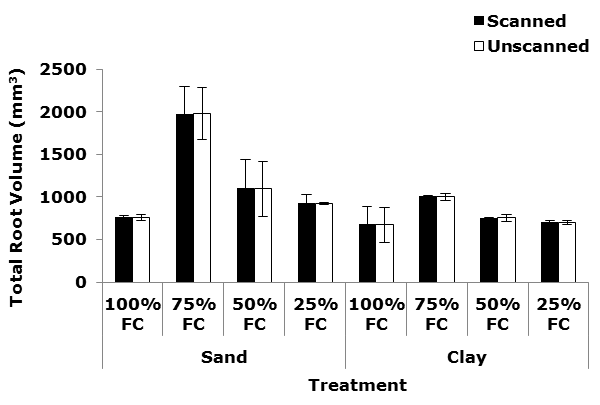


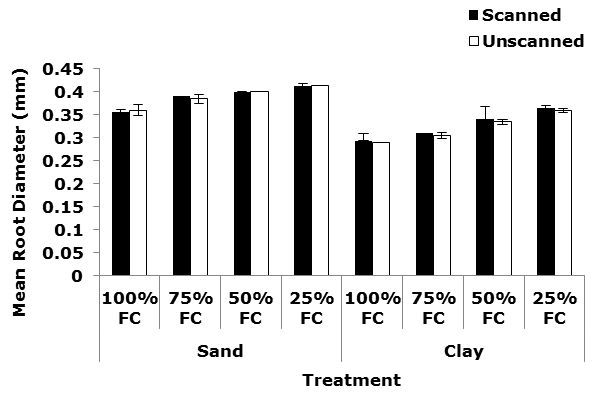


Supplementary Figure 4: Impact of X-ray CT on root growth in wheat variety Zebedee. Zebedee at 21 days was grown under four levels of water stress (100%, 75%, 50% and 25% FC) in sandy loam and clay loam soil types. Bars indicate means ± S.D (n=4).


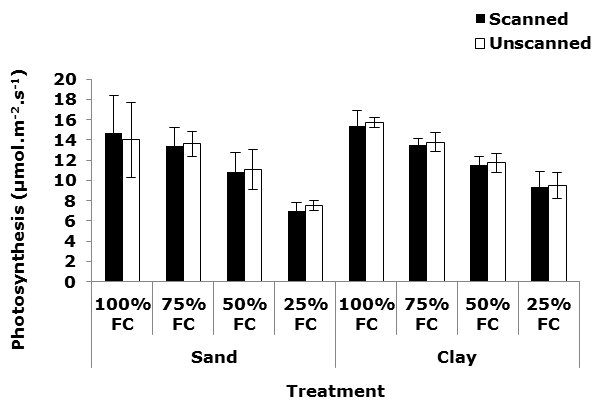


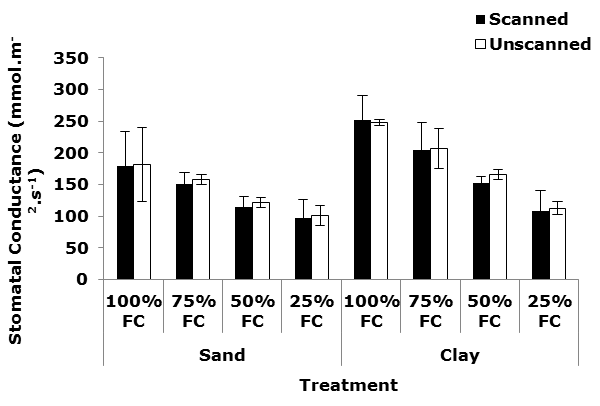


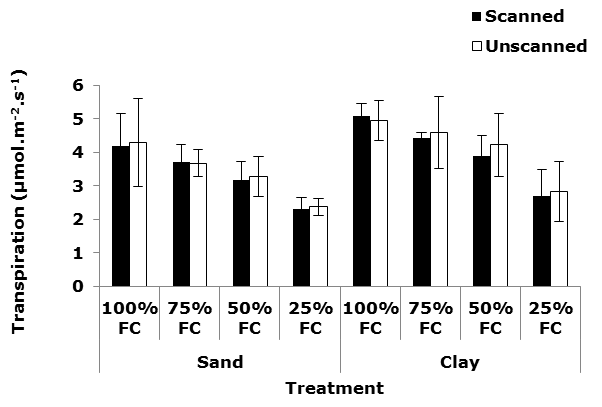


**Supplementary Figure 5:** Impact of X-ray CT on shoot growth in wheat variety Zebedee. Zebedee at 21 days was grown under four levels of water stress (100%, 75%, 50% and 25% FC) in sandy loam and clay loam soil types. Bars indicate means ±S.D (n=4).
